# Integral HIV-STI diagnosis at the first VCT visit as strategy to prevent late ART initiation in the HIV-AIDS program of Mexico City (HIVPMC)

**DOI:** 10.1186/1742-4690-9-S1-P73

**Published:** 2012-05-25

**Authors:** L Juárez-Figueroa, A González-Rodríguez, E Rodríguez-Nolasco

**Affiliations:** 1 HIV Laboratory at HIV-Aids Program of Mexico City, Mexico City, Mexico

## Introduction

HAART and laboratory monitoring of PLWHIV are available in México. Nevertheless, lack of opportune detection impeded early ART initiation, thus favoring HIV transmission and increased incidence of morbidity and mortality due to AIDS conditions before ART starts.

## Methods

On 2010 HIVPMC started faster HIV/STI diagnosis at first VCT visit combining a rapid HIV test and parallel blood analysis with automated HIV 4th generation/STI serology (Abbott Architect). Since fall 2011, initial CD4 counting (Becton Dickinson FacsCalibur) in HIV+ clients, also done at the first visit, favoured a rapid HIV infection staging. On 2012 HIV viral load analysis (Abbott RT-PCR), required in Mexico for ART initiation, was added at the first VCT visit.

## Results

**Figure 1:**
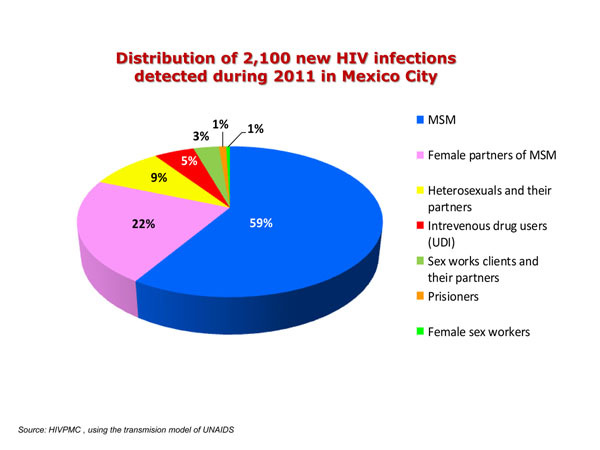


The integral HIV/STI diagnosis at the first VCT visit reduced dramatically the time elapsed before, between the first HIV detection with a rapid test or ELISA and the completion of laboratory studies necessary for starting ART. The attendance of vulnerable groups to VCT at Condesa Clinic scaled up with 51% detection increment during 2011. A 60% of new detected PLWHIV did not return for follow up and treatment as shown by the national HIV data base SALVAR. Figure 1 shows the distribution of new infections on 2011 (UNAIDS model).

## Conclusions

Integral HIV/STI diagnostic self promoted the HIV VCT increasing VCT demand while reducing desertion of number new patients. This model should be expanded to HIV clinics in 31 Mexican States. Depending on the size of the population to be serviced a combination of serial HIV rapid tests, ELISA and simplified point of care CD4 counting could be used.

The quantity of non-returning patients highlights the need of education oriented to people at risk of HIV infection while individual post-test counseling/accompanying could be also individually addressed.

